# Polymer Physics: From Theory to Experimental Applications

**DOI:** 10.3390/polym16060768

**Published:** 2024-03-11

**Authors:** Célio Fernandes, Luís L. Ferrás, Alexandre M. Afonso

**Affiliations:** 1 Center for Studies of Transport Phenomena (CEFT), Department of Mechanical Engineering, Faculty of Engineering, University of Porto, 4200-465 Porto, Portugal; lferras@fe.up.pt (L.L.F.); aafonso@fe.up.pt (A.M.A.); 2 Center of Mathematics (CMAT), University of Minho, Campus of Azurém, 4800-058 Guimarães, Portugal; 3 ALiCE, Associate Laboratory in Chemical Engineering, Faculdade de Engenharia, Universidade do Porto, Rua Dr. Roberto Frias s/n, 4200-465 Porto, Portugal

The significance of polymer processing techniques cannot be overstated in the production of polymer components. The primary objective is to create parts that meet specific quality criteria, typically encompassing mechanical performance, dimensional accuracy, and visual aesthetics. Achieving optimal efficiency in polymer processing requires the use of advanced modeling codes in conjunction with experimental work to understand and optimize the underlying processes. In this editorial, we present cutting-edge papers that contribute to the numerical, theoretical, and experimental knowledge of polymer physics.

Regarding numerical methods, Bertoco et al. [1] developed a novel numerical method that was designed to address three-dimensional unsteady free surface flows incorporating integral viscoelastic constitutive equations, specifically the K–BKZ–PSM (Kaye–Bernstein, Kearsley, Zapas–Papanastasiou, Scriven, and Macosko) model. This new proposed methodology employs a second-order finite difference approach along with the deformation fields method to solve the integral constitutive equation and the marker particle method (known as marker-and-cell) to accurately capture the evolution of the fluid’s free surface. This newly developed numerical method proved its effectiveness in handling complex fluid flow scenarios, including confined flows and extrudate swell simulations of Boger fluids. Larsen et al. [2] presented a non-isothermal, non-Newtonian Computational Fluid Dynamics model for the mixing of a highly viscous polymer suspension in a partially filled sigma blade mixer. This model accounts for viscous heating and the free surface of the suspension. The rheological model was found by calibration with experimental temperature measurements and was exploited to study the effect of applying heat both before and during mixing on the suspension’s mixing quality. Two mixing indexes were used to evaluate the mixing condition, namely, the Ica Manas-Zloczower dispersive index and Kramer’s distributive index. The Kramer index results were stable and indicated that the particles in the suspension could be well distributed. Interestingly, the results highlighted that the speed at which the suspension becomes well distributed is almost independent of applying heat both before and during the process. Rusková et al. [3] performed coarse-grained molecular dynamics simulations of DNA polymers pushed inside infinite open chiral and achiral channels. They investigated the behavior of the polymer metrics in terms of span, monomer distributions, and changes in the topological state of the polymer in the channels. These authors also compared the regime of pushing a polymer inside the infinite channel to the case of polymer compression in the finite channels of knot factories investigated in earlier works. It was observed that the compression in the open channels affects the polymer metrics to different extents in the chiral and achiral channels. Also, the chiral channels give rise to the formation of equichiral knots with the same handedness as the chiral channels.On the theoretical side, Nitta et al. [4] studied the effects of annealing time on the specific heat enthalpy of polystyrene above the glass transition temperature. These authors extended the Tool–Narayanaswamy–Moynihan model to describe the endothermic overshoot peaks through the dynamic mechanical spectra. In their work, these authors consider the viewpoint that the enthalpy recovery behavior of glassy polystyrene (PS) has a common structural relaxation mode with linear viscoelastic behavior. As a consequence, the retardation spectrum evaluated from the dynamic mechanical spectra around the primary Tg peak was used as the recovery function of the endothermic overshoot of specific heat. In addition, the sub-Tg shoulder peak around the Tg peak was found to be related to the structural relaxation estimated from light scattering measurements. The enthalpy recovery of annealed PS was quantitatively described using retardation spectra of the primary Tg as well as the kinetic process of the sub-Tg relaxation process. Baranovskii [5] studied the unidirectional pressure-driven flow of a second-grade fluid within a planar channel bounded by impermeable solid walls. These authors examined the well-posed nature of this problem and derived its analytical solution while imposing weak regularity conditions on a function representing the initial velocity distribution. In addition, the notion of a generalized solution, defined as the limit of a uniformly convergent sequence of classical solutions with diminishing perturbations in the initial data, was employed, and the unique solvability of the problem under consideration in the class of generalized solutions was achieved. The conclusion of this work was that the developed analytical solutions facilitate a deeper comprehension of the qualitative characteristics of time-dependent flows involving polymer fluids.

Concerning experimental work, Alsarkhi et al. [6] presented a comprehensive experimental investigation concerning the effect of drag-reducing polymers (DRPs) on enhancing the throughput and reducing the pressure drop for a horizontal pipe carrying a two-phase flow of an air and water mixture. They showed the ability of these polymer entanglements to dampen turbulence waves and change the flow regime, and it was observed that the maximum drag reduction always occurs when the highly fluctuating waves are effectively reduced by DRPs. Furthermore, different empirical correlations have been developed that improve the ability to predict the pressure drop after the addition of DRP. The correlations showed low discrepancies for a wide range of water and air flow rates. Ham et al. [7] investigated the effects of the number of InO_x_ SIS (sequential infiltration synthesis) cycles on the chemical and electrochemical properties of PANI-InO_x_ thin films via combined characterization using X-ray photoelectron spectroscopy, ultraviolet–visible spectroscopy, Raman spectroscopy, Fourier-transform infrared spectroscopy, and cyclic voltammetry. The area-specific capacitance values of PANI-InO_x_ samples prepared with 10, 20, 50, and 100 SIS cycles were 1.1, 0.8, 1.4, and 0.96 mF/cm^2^, respectively. These results highlighted that the formation of an enlarged PANI-InO_x_ mixed region directly exposed to the electrolyte is key to enhancing the pseudocapacitive properties of the composite films. Ahn et al. [8] fabricated a cellulose nanocrystal (CNC)-embedded aerogel-like chitosan foam and carbonized the 3D foam for electrical energy harvesting. The nanocrystal-supported cellulose foam showed a high surface area and porosity, homogeneous size ranging from various microscales, and a high quality of absorbing external additives. In order to prepare the CNC, microcrystalline cellulose (MCC) was chemically treated with sulfuric acid. The CNC incorporates into chitosan, enhancing mechanical properties, crystallization, and the generation of the aerogel-like porous structure. The weight percentage of the CNC was 2 wt% in the chitosan composite. The CNC/chitosan foam was produced using the freeze-drying method, and the CNC-embedded CNC/chitosan foam was carbonized. These authors found that the degree of crystallization of carbon structure increased, including the CNCs. Both CNC and chitosan are degradable materials when CNC includes chitosan, which can form a high surface area with some typical surface-related morphology. The electrical cyclic voltametric results indicated that the vertical composite specimen had superior electrochemical properties compared to the horizontal composite specimen. In addition, the BET measurement indicated that the CNC/chitosan foam possessed a high porosity, especially mesopores with layer structures. At the same time, the carbonized CNC led to a significant increase in the portion of micropore. Xing et al. [9] experimentally studied the dynamic crushing performance of expanded polyethylene (EPE) and analyzed the influence of thickness and dropping height on its mechanical behavior based on the stress–energy method. Hence, a series of impact tests were carried out on EPE foams with different thicknesses and dropping heights. The maximum acceleration, static stress, dynamic stress, and dynamic energy of EPE specimens were obtained through a dynamic impact test. Then, according to the principle of the stress–energy method, the functional relationship between dynamic stress and dynamic energy was obtained through exponential fitting and polynomial fitting, and the cushion material constants a, b, and c were determined. When analyzing the influence of thickness and dropping height on the dynamic cushioning performance curves of EPE, it was found that at the same drop height, with the increase of thickness, the opening of the curve gradually becomes larger. The minimum point on the maximum acceleration–static stress curve also decreased with the increase in thickness. When the dropping height was 400 mm, compared to the foam with a thickness of 60 mm, the tested maximum acceleration value of the lowest point of the specimen with a thickness of 40 mm increased by 45.3%, and the static stress was both 5.5 kPa. When the thickness of the specimen was 50 mm, compared to the dropping height of 300 mm, the tested maximum acceleration value of the lowest point of the specimen with a dropping height of 600 mm increased by 93.3%. Therefore, the dynamic cushioning performance curve of EPE foams can be quickly obtained by the stress–energy method when the precision requirement is not high, which provides a theoretical basis for the design of cushion packaging. Harichane et al. [10] studied the influence of three types of PCEs (polycarboxylate ether superplasticizer), which all have different molecular architectures, on the rheological and mechanical behavior of high-performance concretes containing 10% SF (silica fume) as a partial replacement of cement. Their results revealed that the carboxylic density of PCE has an influence on its compatibility with SF.

Finally, Subbotin et al. [11] presented a review devoted to understanding the role of elasticity in the main flow modes of polymeric viscoelastic liquids—shearing and extension. The flow through short capillaries is the central topic for discussing the input of elasticity to the effects, which are especially interesting for shear. An analysis of the experimental data made it possible to show that the energy losses in such flows are determined by the Deborah and Weissenberg numbers. These criteria are responsible for abnormally high entrance effects as well as for mechanical losses in short capillaries. In addition, the Weissenberg number determines the threshold of the flow instability due to the liquid-to-solid transition. In extension, this criterion shows whether deformation takes place as flow or as elastic strain. However, the stability of a free jet in extension not only depends on the viscoelastic properties of a polymeric substance but also on the driving forces: gravity, surface tension, etc.

The editors express confidence that this editorial will facilitate researchers in comprehending the fundamental principles of polymer physics from both numerical and experimental viewpoints. Moreover, this editorial serves as a valuable reference for those keen on staying abreast of cutting-edge technologies.
